# Trends, structural changes, and assessment of time series models for forecasting hospital discharge due to death at a Mexican tertiary care hospital

**DOI:** 10.1371/journal.pone.0248277

**Published:** 2021-03-08

**Authors:** Edel Rafael Rodea-Montero, Rodolfo Guardado-Mendoza, Brenda Jesús Rodríguez-Alcántar, Jesús Rubén Rodríguez-Nuñez, Carlos Alberto Núñez-Colín, Lina Sofía Palacio-Mejía

**Affiliations:** 1 Department of Research, Hospital Regional de Alta Especialidad del Bajío, León, Guanajuato, México; 2 Programa de Biotecnología, Universidad de Guanajuato, Celaya, Guanajuato, México; 3 CONACYT-Instituto Nacional de Salud Pública, Cuernavaca, Morelos, México; South China University of Technology, CHINA

## Abstract

**Background:**

Data on hospital discharges can be used as a valuable instrument for hospital planning and management. The quantification of deaths can be considered a measure of the effectiveness of hospital intervention, and a high percentage of hospital discharges due to death can be associated with deficiencies in the quality of hospital care.

**Objective:**

To determine the overall percentage of hospital discharges due to death in a Mexican tertiary care hospital from its opening, to describe the characteristics of the time series generated from the monthly percentage of hospital discharges due to death and to make and evaluate predictions.

**Methods:**

This was a retrospective study involving the medical records of 81,083 patients who were discharged from a tertiary care hospital from April 2007 to December 2019 (first 153 months of operation). The records of the first 129 months (April 2007 to December 2017) were used for the analysis and construction of the models (training dataset). In addition, the records of the last 24 months (January 2018 to December 2019) were used to evaluate the predictions made (test dataset). Structural change was identified (Chow test), ARIMA models were adjusted, predictions were estimated with and without considering the structural change, and predictions were evaluated using error indices (MAE, RMSE, MAPE, and MASE).

**Results:**

The total percentage of discharges due to death was 3.41%. A structural change was observed in the time series (March 2009, p>0.001), and ARIMA(0,0,0)(1,1,2)_12_ with drift models were adjusted with and without consideration of the structural change. The error metrics favored the model that did not consider the structural change (MAE = 0.63, RMSE = 0.81, MAPE = 25.89%, and MASE = 0.65).

**Conclusion:**

Our study suggests that the ARIMA models are an adequate tool for future monitoring of the monthly percentage of hospital discharges due to death, allowing us to detect observations that depart from the described trend and identify future structural changes.

## Introduction

Data on hospital discharges due to death can be used as a valuable instrument for hospital planning and management [1]. In Mexico, the General Directorate of Health Information (*Dirección General de Información en Salud*—DGIS; Spanish acronym) is the operational body of the Ministry of Health (*Secretaría de Salud*—SSA; Spanish acronym) that is responsible for generating statistics on health and has various information subsystems, including the Automated Hospital Discharge System (*Sistema Automatizado de Egresos Hospitalarios*–SAEH; Spanish acronym) [2]. The number of national hospital discharges recorded by Mexican SSA hospitals as primary sources in the SAEH is estimated to be 3 million cases per year, and of these, approximately 2% are deaths [3]. There are descriptive reports on national and regional hospital discharges [2,4], but there are few inferential studies on trends, the identification of associated variables, or predictions about such hospital discharges.

The Bajío Regional Hospital of High Specialty (*Hospital Regional de Alta Especialidad del Bajío*—HRAEB) has provided clinical, diagnostic, and tertiary treatment services since April 2007. It has 184 census beds, does not have an emergency department, and discharges patients with complex pathologies. Its source documents for recording hospital discharges are the clinical file and death certificate. The corresponding data from on these documents are captured in the computer platform of the SAEH by the Medical Statistics area personnel of the HRAEB according to the guidelines of the DGIS [5].

Since 1986, the Health Care Financing Administration has incorporated the hospital mortality rate as a qualitative comparator of American hospitals, which has encouraged the use of hospital care outcome indicators across the world [6]. Hospital mortality is one of the most frequently used indicators of quality of care [7], and the quantification of deaths can be considered a measure of the effectiveness of hospital intervention, although it should not be forgotten that this indicator is influenced by other factors, such as the pathology being treated, population structure and accessibility to the hospital unit [8]. A high percentage of hospital discharges due to death can be associated with deficiencies in the quality of hospital care [9].

Since in this case the percentage of discharges due to death it is a change of percentage over time, it can be defined as a time series. To model this mortality series, linear models are typically constructed [10]. Some studies have even analyzed the trends of hospital discharges for a specific cause [11], and other authors have estimated predictions [12]. Usually Autoregressive integrated moving average (ARIMA) models are used for analyzing and forecasting time series data and are also capable of modelling a wide range of seasonal data [13–15]. The Box-Jenkins (ARIMA) econometric modelling is a forecasting technique that completely ignores independent variables in making forecast. It takes into account historical data and decomposes it into Autoregressive (AR) process, where there is a memory of past events; an Integrated (I) process, which accounts for stabilizing or making the data stationary, making it easier to forecast; and a Moving Average (MA) of the forecast errors, such that the longer the historical data, the more accurate the forecasts will be, as it learns over time [16,17].

For the identification of the ARIMA model, two goodness-of-fit statistics that are most commonly used for the model selection are; Akaike Information Criterion (AIC) and Schwarz Bayesian Information Criterion (BIC). The AIC and BIC are determined based on a likelihood function [15]. When comparing two models, the one with the lower AIC or BIC is generally better [18]. Low AIC or BIC values suggest that a model nicely straddle the requirements of goodness-of-fit and parsimony [19].

In addition, regarding hospital deaths, there is scientific evidence that certain events, including enabling hospital admissions on weekends [20,21], varying the number and educational level of nursing staff as well as the ratio of nurses per census bed [22–25], increasing the number of hospitalized patients [26,27] and increasing the volume of surgical patients [28,29], can increase the risk of hospital mortality and therefore raise the percentage of hospital discharges due to death.

The aim of this study was to determine the overall percentage of hospital discharges due to death in a Mexican tertiary care hospital from its opening in April 2007 to December 2019, to describe the characteristics of the time series generated from the monthly percentage of hospital discharges due to death from April 2007 to December 2017 (first 129 months of operation) and to make and evaluate predictions of the monthly percentage of hospital discharges due to death from January 2018 to December 2019 (24 months later).

## Materials and methods

### Patients

This retrospective study of Hispanic-Mexican patients included all the records of patients (n = 81,083) who were discharged from a tertiary care hospital (HRAEB) located in the city of León in the state of Guanajuato, Mexico, from its opening in April 2007 to December 2019 (153 months of operation). The dataset is secondary and was obtained from the national government records of the SAEH subsystem operated by DGIS [3]. This is a subsystem that compiles national hospital discharge information from Mexican hospitals as primary sources. The dataset was divided into two parts. The records of the first 129 months (April 2007 to December 2017) were used for the analysis and construction of the models (training dataset). In addition, the records of the last 24 months (January 2018 to December 2019) were used to evaluate the predictions made (test dataset).

### Ethical considerations

The protocol of this study was reviewed and approved by the Institutional Research and Ethics Committees of the HRAEB and the National Institute of Public Health (*Instituto Nacional de Salud Pública*) of Mexico (approval numbers: CI/HRAEB/2019/046 and PT 211, respectively).

### Statistical analysis

All data were analyzed with the statistical software R [30]. Initially, a descriptive analysis was implemented, and the time series associated with the monthly percentage of hospital discharges due to death was plotted. For the time series, the trend was modeled using a simple linear regression model using least squares (SLRMLS) without segmentation [10]. The assumptions of normality, homoscedasticity, and independence of the residuals were verified using the Kolmogorov-Smirnov, White, and Durbin-Watson tests, respectively. Next, the instances where possible structural changes could occur (sudden changes in the trend of the time series) were identified using the Chow test [31] with the statistical R package “strucchange”, which is designed to test structural changes in linear regression models [32]. Next, additive decomposition of the series into its components was performed as follows: trend, seasonal variations (seasonality), and irregular fluctuations (random) [10]. ARIMA models were used. The name of this procedure comes from its three components: autoregression (AR), integration (I), and moving average (MA). The ARIMA(p, d, q) model is fitted to the observed data, where p, d and q represent the order of the three respective components. Regarding the integrated components of the ARIMA model, if the stochastic process associated with a time series has a unit root, we can conclude that it is a nonstationary time series. Therefore, the Dickey-Fuller test was implemented for each of the y_t_ series to determine if it was stationary. If not, we proceeded to differentiate each series (generating a new series *z_t_* = *y_t_*−*y*_*t*−1_ with *t* = 2,3…,*n*) as many times as necessary (d) to obtain a stationary time series and thus conclude that the original series y_t_ was integrated of order (d). In addition, autocorrelation (ACF) and partial autocorrelation (PACF) plots were constructed for each series and for those series that resulted from differentiation [33] to empirically confirm whether the series could be considered stationary and identify the possible number of lags for each of the remaining components of the ARIMA model.

In order to be able to predict the monthly percentage of discharges due to death, with and without considering the structural change identified in the series, for the forecasting, the seasonal ARIMA models were adjusted. The seasonal ARIMA(p, d, q)(P, D, Q)_s_ process is given by Φ(B^s^)ϕ(B)(1−B^s^)^D^(1−B)^d^y_t_ = c+Θ(B^s^)θ(B)ϵ_t_ where Φ(z) and Θ(z) are polynomials of orders P and Q respectively, each containing no roots inside the unit circle. If c≠0, there is an implied polynomial of order d + D in the forecast function [34]. The selection of the proper seasonal ARIMA model in each case was done by the auto.arima function from the R package “forecast” [35]. This function returns the best ARIMA model according to the AIC or BIC value [36]. The function conducts a search over a range of possible models within the order constraints and picks the most suitable one [37]. The auto.arima function is based mainly on the Hyndman–Khandakar algorithm, which combines unit element tests, minimization of corrected Akaike tests (AICc), and the maximum likelihood method to obtain the model most appropriate for the data. The criteria of goodness-of-fit, based on the information criterion, were taken into account [38]: *AIC* = −2ln(*L*)+2(*p+q+P+Q+k*) and *BIC* = −2ln(*L*)+(*p+q+P+Q+k*)ln(*n*) where k = 1 if c≠0 and 0 otherwise, and *L* is the maximized likelihood of the model fitted to the differenced data (1−*B^s^*)^*D*^(1−*B*)^*d*^ y_*t*_. After selecting the models for each series, it was verified that the residuals did not have a dependence structure and that they followed a white noise process.

Finally, predictions were made (point forecasts and prediction intervals) of the series of the percentage of discharges due to death, with and without considering the structural change identified in the series using the function forecast from the R package “forecast” [35]. These predictions were evaluated using four error indices: two scale-dependent error indices: mean absolute error (MAE) and root mean square error (RMSE); one percentage error index: mean absolute percentage error (MAPE); and one scale-free error index: mean absolute scaled error (MASE) [39–42]. In all tests, the significance level of α = 0.05 was used.

## Results

A total of 81,083 hospital discharges, of which 2,767 were deaths, were generated in the HRAEB from April 2007 to December 2019 and included in the final analysis of this study. **Table 1** describes the total hospital discharges of the HRAEB per year, and a total percentage of discharges due to death of 3.41% was observed. In 2009, the highest annual percentage of hospital discharges due to death, 5.77%, was recorded. In contrast, 2018 had the lowest annual percentage of hospital discharges due to death, 2.50%.

**Table 1:** Hospital discharges in the HRAEB grouped by year.

Year	Number of hospital discharges	Number of hospital discharges due to death	% Hospital discharges due to death
2007 (Apr-Dec)	623	29	4.65%
2008	3,045	146	4.79%
2009	5,063	292	5.77%
2010	5,443	257	4.72%
2011	5,464	244	4.47%
2012	5,884	201	3.42%
2013	6,361	206	3.24%
2014	7,246	198	2.73%
2015	7,867	240	3.05%
2016	8,555	225	2.63%
2017	8,949	289	3.23%
2018	8,843	221	2.50%
2019	7,740	219	2.83%
Total	81,083	2767	3.41%

**Fig 1:**
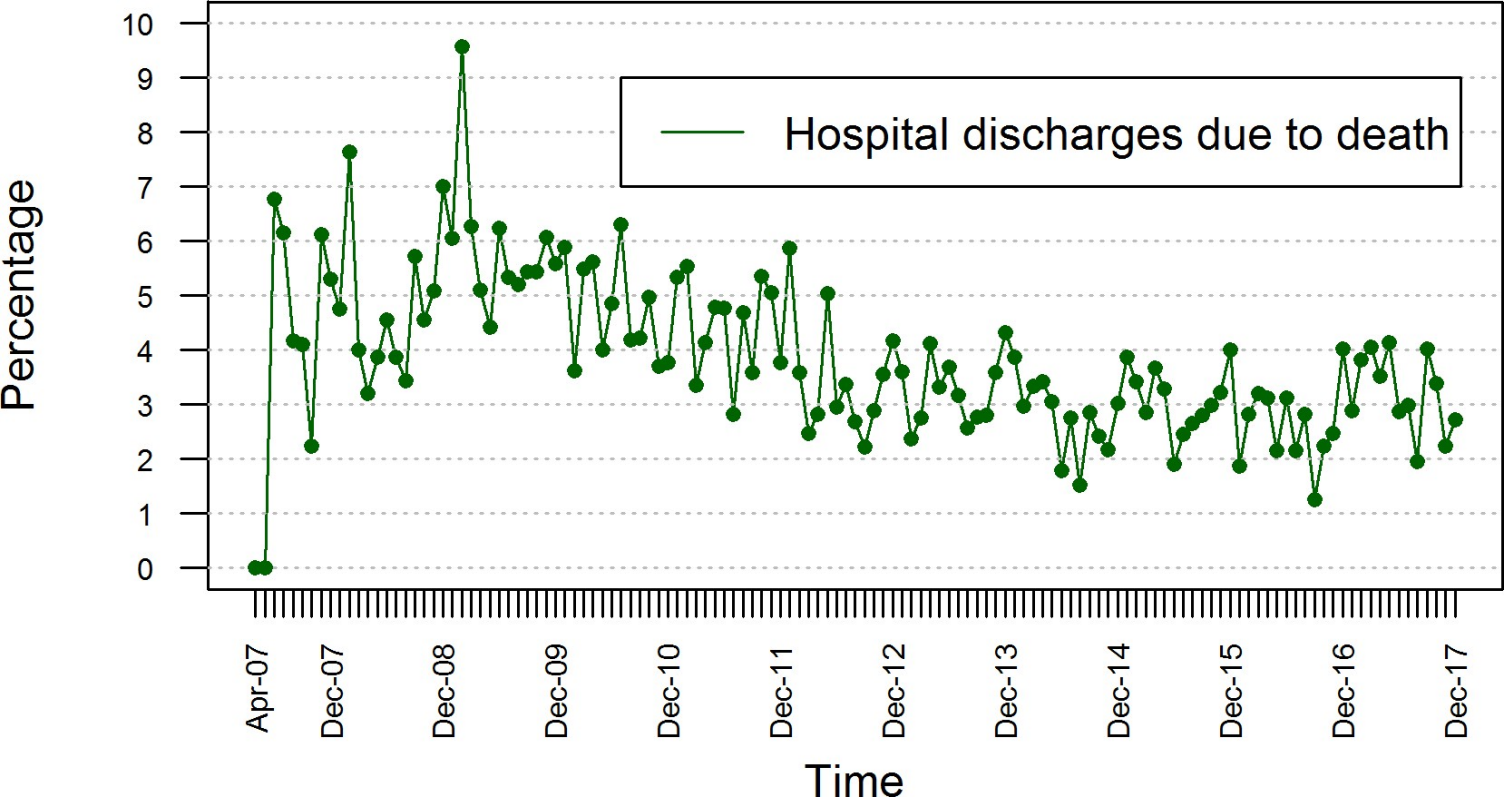
Time series of the monthly percentages of HRAEB hospital discharges due to death.

**Fig 1** shows the time series of the monthly percentage of hospital discharges due to death in the HRAEB from April 2007 to December 2017. The series consists of 129 observations that oscillate between 0 and 10%, reaching their maximum at t = 23 (February 2009): 9.57%.

**Fig 2:**
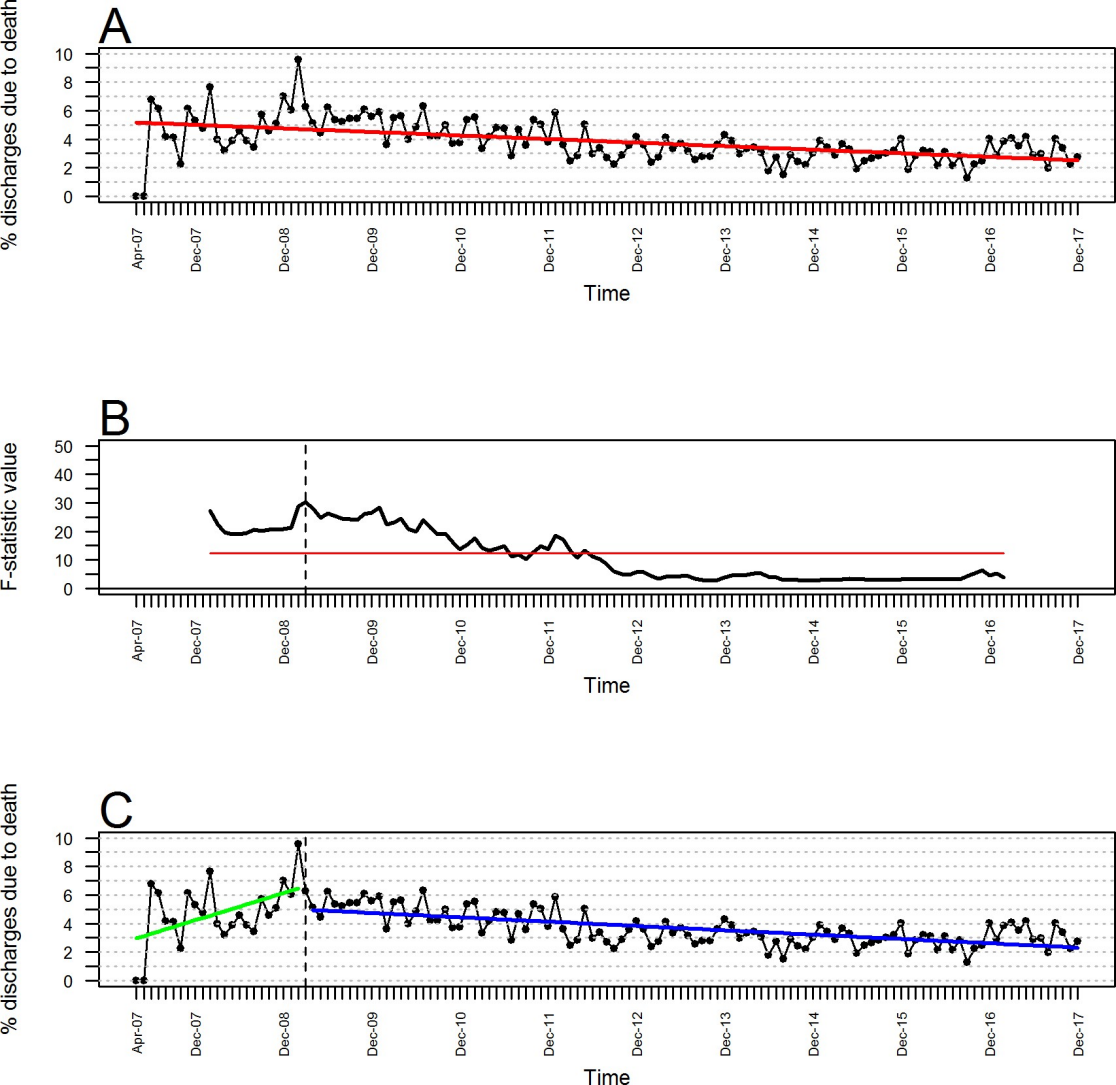
Trends and structural change in the monthly percentage of HRAEB hospital discharges due to death. (A) Trend modeling using a linear model. (B) F-statistics from the Chow test (alpha = 0.05; red horizontal line). (C) Segmented trend modeling using two linear models.

**Fig 2A** shows the graph of the estimated SLRMLS to model the trend of the percentage of hospital discharges due to death with respect to time, with R^2^ = 28.24.%, slope = -0.021 (standard error (SE) = 0.003), and y-intercept = 5.182 (SE = 0.220). This model describes a trend with an apparent decrease in the percentage of hospital discharges due to death over time, but when verifying the assumptions of the model, it was observed that the residuals did not meet the assumption of independence (p < 0.001). This was done through the implementation of the Durbin-Watson test, which resulted in the detection of positive autocorrelation. Next, **Fig 2B** graphically illustrates the statistics associated with the Chow tests, which we performed to detect the possible instances where there was a structural change in the trend of the time series. There was a structural change at (March 2009) at the level. Finally, **Fig 2C** shows the graphs of the two estimated models (SLRMLS) that resulted from modeling the trend of the percentage of hospital discharges due to death with respect to time considering the structural change identified at t = 24. The first model describes the observations before t = 24; it has R^2^ = 24.00%, a slope of 0.159 (SE = 0.062), and a y-intercept of 2.804 (SE = 0.845). This model shows an increasing trend before t = 24. The second model describes the observations after t = 24; it has R^2^ = 44.46%, a slope of -0.025 (SE = 0.003) and a y-intercept of 5.565 (SE = 0.230). This model shows a decreasing trend after t = 24. Both models together show a significantly lower combined sum of squared estimate of errors (SSE; SSE_1_+ SSE_2_) than the SSE resulting from the linear-trend SLRMLS that described the decreasing trend in **Fig 2A** (p < 0.001).

**Fig 3:**
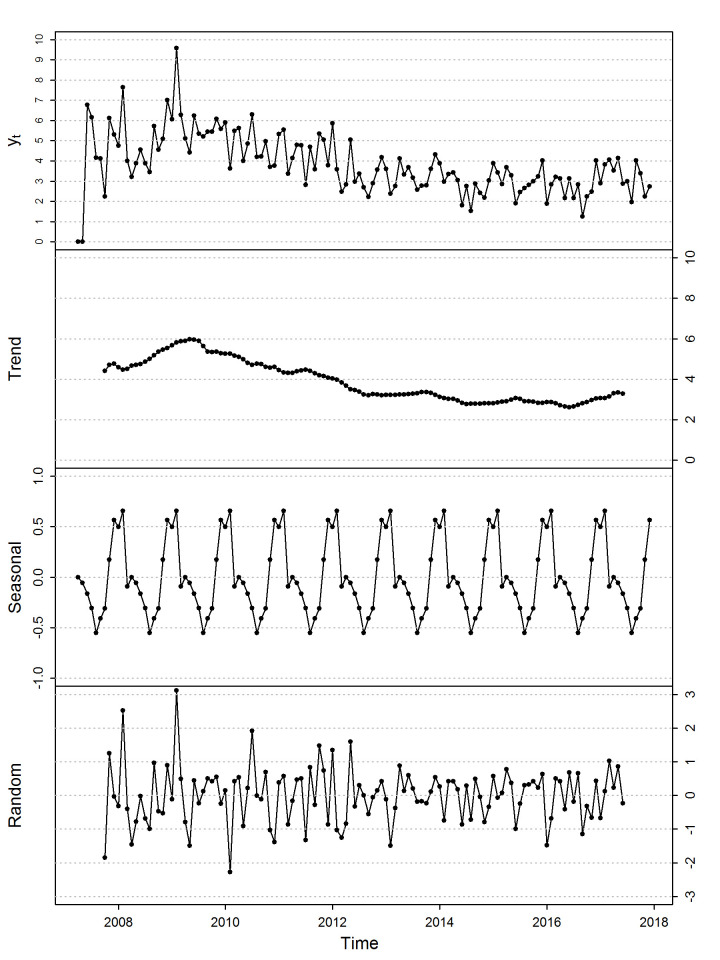
Additive decomposition of the monthly percentage of HRAEB hospital discharges due to death.

**Fig 3** shows the exploratory graph of the additive decomposition of the series of the monthly percentage of HRAEB hospital discharges due to death as a trend, from seasonal variations (seasonality), and from irregular fluctuations (random). An apparent increasing trend was observed from the beginning until mid-2009, and later, there was an apparent decreasing trend. There was also an apparent seasonal variation with period s = 12.

In trying to verify whether the series of the monthly percentage of hospital discharges due to death was stationary, the presence of a unit root could be identified (Dickey-Fuller = -3.4712, lag order = 5, p = 0.48), from which we could presume that this was a nonstationary time series. When differentiating the series, we concluded that the series y_t_ was integrated of order D = 1, since it was necessary to differentiate the series once to obtain the series (y_t_-y_t-1_) that was stationary (Dickey-Fuller = -6.7907, lag order = 5, p <0.01).

**Fig 4:**
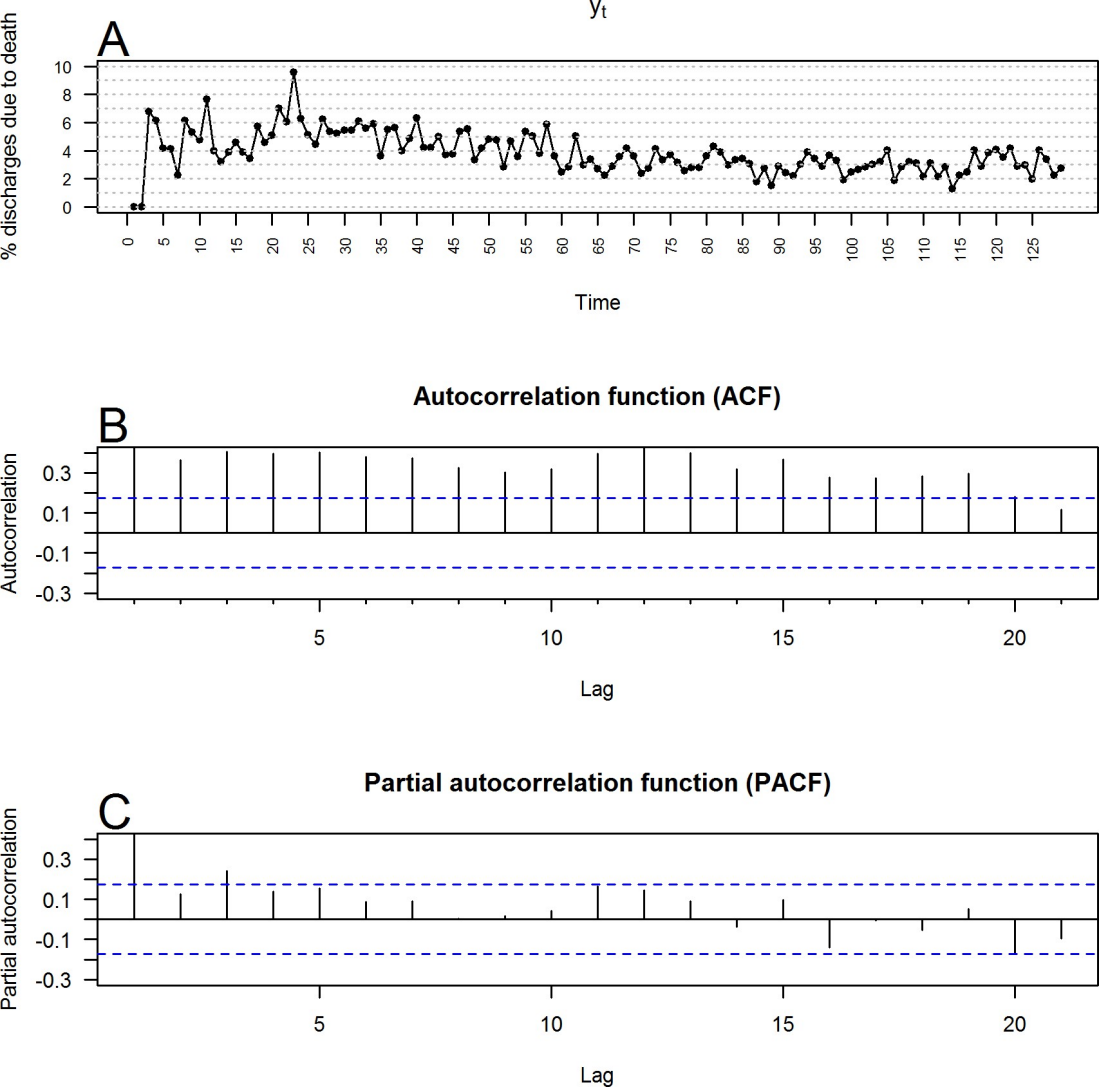
Monthly percentage of HRAEB hospital discharges due to death. (A) Original values. (B) Autocorrelation function (alpha = 0.05; blue bands). (C) Partial autocorrelation function (alpha = 0.05; blue bands).

**Fig 5:**
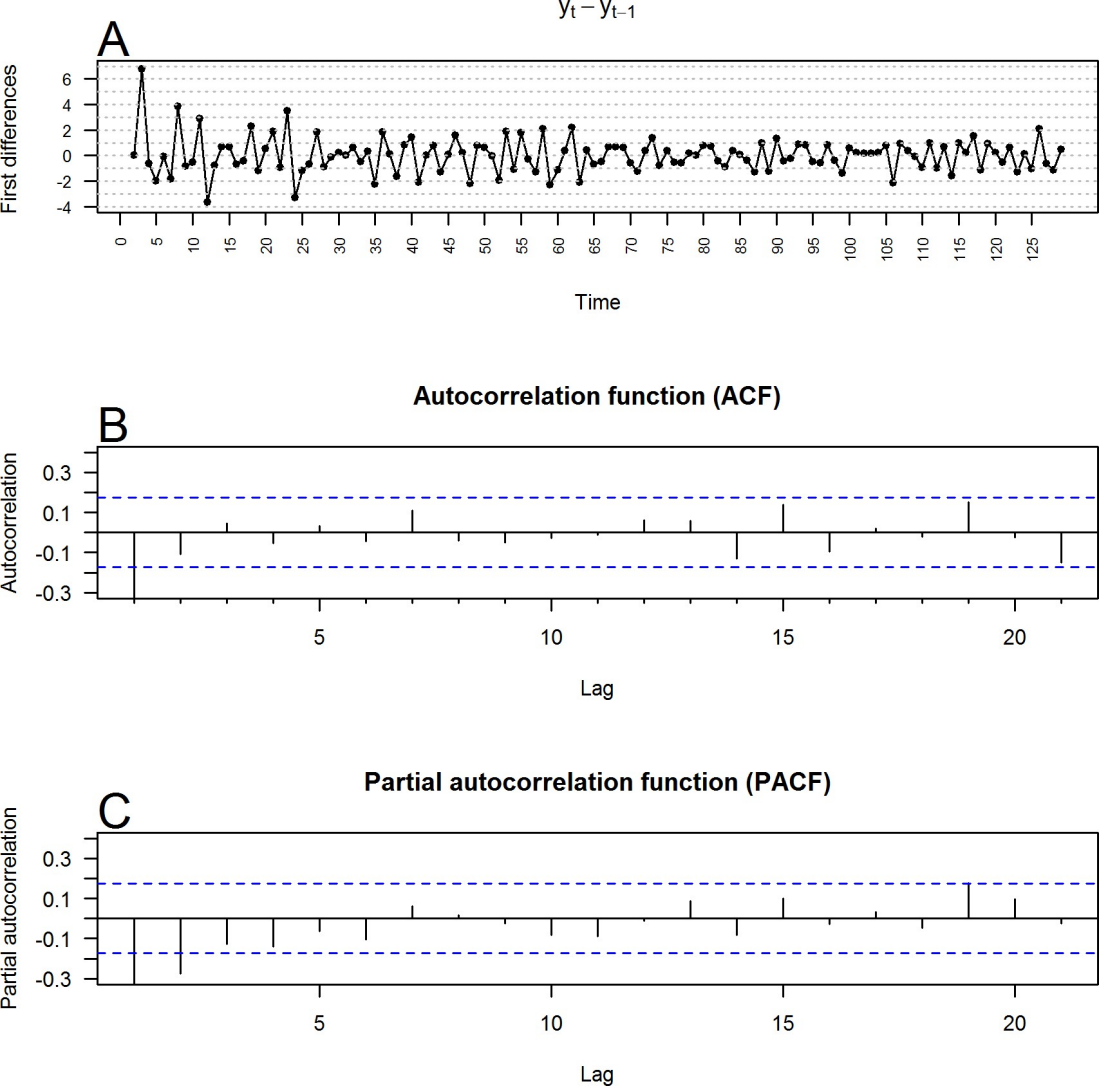
First differences in the monthly percentage of HRAEB hospital discharges due to death. (A) First differences. (B) Autocorrelation function (alpha = 0.05; blue bands). (C) Partial autocorrelation function (alpha = 0.05; blue bands).

**Fig 4A** shows the monthly series of hospital discharges due to death at the HRAEB (y_t_). **Fig 4B** and **Fig 4C** show the autocorrelation (ACF) and partial autocorrelation (PACF) functions of the time series, respectively. The ACF function of the values of the time series was cut with extreme slowness, which reaffirmed that the series was nonstationary. Similarly, **Fig 5A** shows the differentiated series (y_t_-y_t-1_), and **Fig 5B** and **5C** show the ACF and PACF of the differentiated series, respectively. They were cut after the second phase, which reaffirmed that the differentiated series was stationary. In addition, the analysis of both figures empirically suggested that p = 1 or 2 and q = 1 or 2 may be relevant orders of the AR and MA components of an ARIMA model for the series studied. After confirming that the series of the monthly percentage of discharges due to death at the HRAEB could be modeled with a seasonal ARIMA model with integrated order D = 1, the AIC and BIC were calculated in models I (1), AR (0), AR (1), AR (2), MA (0), MA (1), and MA (2). The number of lags that minimized them occurred in the ARIMA(0,0,0)(1,1,2)_12_ model, that is, when P = 1,Q = 2.

Based on the above, a seasonal ARIMA(0,0,0)(1,1,2)_12_ with drift was adjusted to model the series of the monthly percentage of hospital discharges from HRAEB due to death without considering structural change. The coefficient associated with the AR component (1) was sar1 = 0.632 (SE = 0.455), the coefficients associated with the MA component (2) were sma1 = -1.116 (SE = 0.475) and sma2 = 0.231 (SE = 0.280), and the drift coefficient was -0.017 (SE = 0.005). The AIC and BIC of this model were 450.52 and 419.33, respectively.

For the series of the monthly percentage of HRAEB hospital discharges due to death considering structural change, a seasonal ARIMA(0,0,0)(1,1,2)_12_ with drift model was adjusted. The coefficient associated with the AR (1) component was sar1 = -0.991 (SE = 0.116), the coefficients associated with the MA component (2) were sma1 = 0.289 (SE = 0.275) and sma2 = -0.656 (SE = 0.251), and the drift coefficient was -0.025 (SE = 0.004). The AIC and BIC of this model were 270.64 and 283.3, respectively.

**Fig 6:**
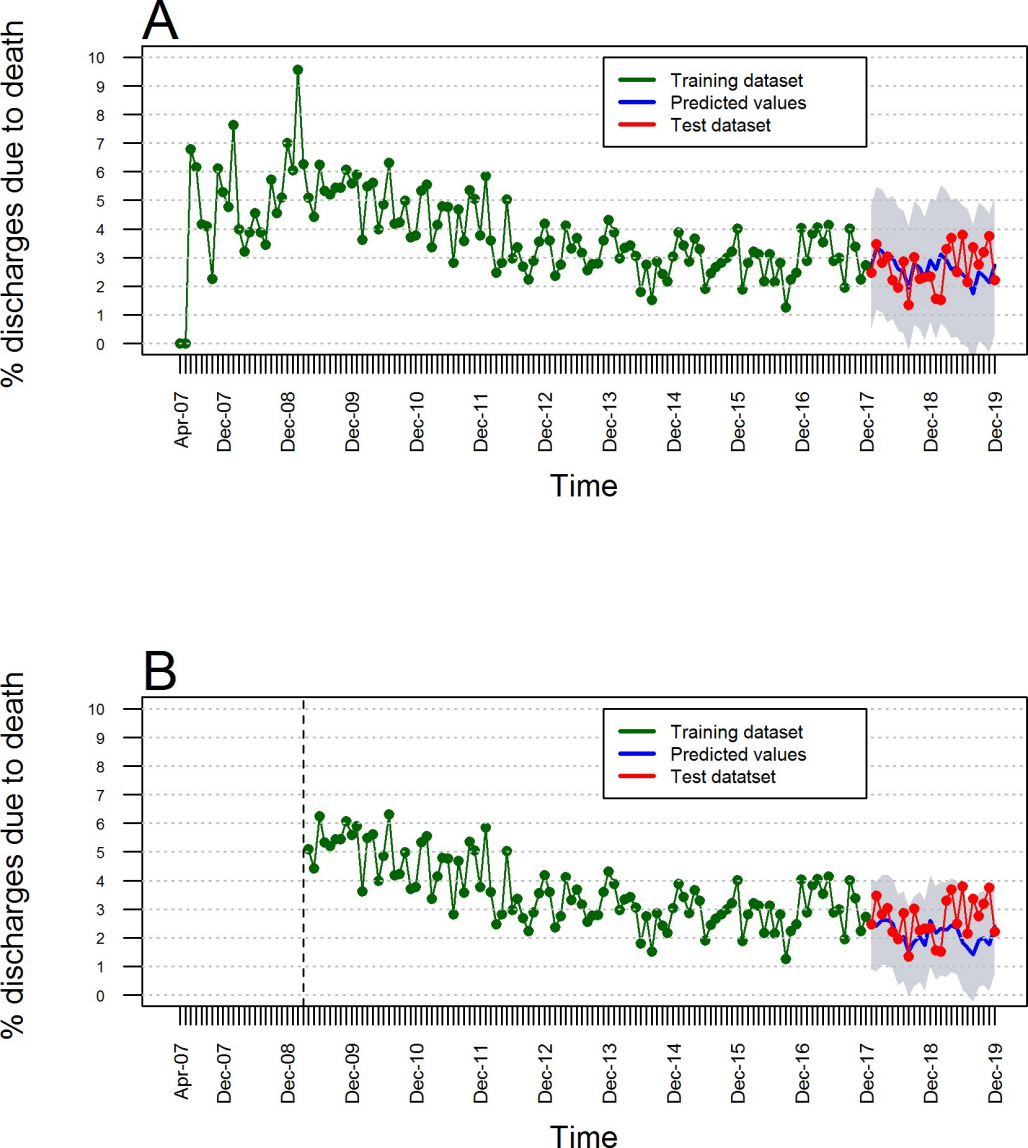
Forecasts from ARIMA(0,0,0)(1,1,2)_12_ models with 90% prediction intervals. (A) Estimated predictions considering no structural change. (B) Estimated predictions considering the identified structural change at t = 24.

**Fig 6A** shows the point predictions with prediction intervals of 90% of the percentage of hospital discharges due to death from the HRAEB for the 24 months included in the test dataset (January 2018 to December 2019) that resulted from adjusting the ARIMA(0,0,0)(1,1,2)_12_ with drift, without considering a structural change. **Fig 6B** shows the predictions for the same period with prediction intervals of 90% that resulted with an ARIMA(0,0,0)(1,1,2)_12_ model with drift but considering the structural change identified at t = 24. When comparing both predictions, the 90% prediction intervals that considered the structural change in the trend were narrower.

**Table 2** shows the predictions for the monthly percentage of hospital discharges due to death from January 2018 to December 2019 considering the two models (no structural change and considering the structural change in the trend). The model that did not consider structural change estimated an average point prediction of the overall percentage of hospital discharges due to death of 2.62 ± 0.39% (range 1.73–3.32%) for the period from January 2018 to December 2019, a percentage higher than that estimated with the prediction average point generated by the model that incorporated the identified structural change 2.12 ± 0.36% (range 1.41–2.62%) for the same period. More accurate estimates (narrower prediction intervals) were obtained when using the model that incorporated the identified structural change, but compared with the actual values observed for the monthly percentage of hospital discharges due to death of 2.66 ± 0.71% (range 1.35–3.79%), very rough point estimates were observed in both cases. However, they were underestimated by the model that considered structural change.

**Table 2:** Predictions and observed values (test dataset) of the monthly percentage of HRAEB hospital discharges due to death.

t	Month	Model not considering structural change			Model considering the identified structural change (t = 24)			Observed values (test dataset)
		ARIMA(0,0,0)(1,1,2)_12_			ARIMA(0,0,0)(1,1,2)_12_			
		Prediction	90% prediction Interval		Prediction	90% prediction Interval		
130	Jan-18	2.70	0.55	4.85	2.48	0.90	4.05	**2.48**
131	Feb-18	3.32	1.17	5.47	2.40	0.82	3.98	**3.48**
132	Mar-18	3.23	1.08	5.38	2.61	1.04	4.19	**2.82**
133	Apr-18	2.89	0.74	5.04	2.62	1.04	4.20	**3.03**
134	May-18	3.01	0.86	5.16	2.51	0.93	4.09	**2.20**
135	Jun-18	2.60	0.45	4.74	1.93	0.35	3.51	**1.95**
136	Jul-18	2.49	0.34	4.64	2.05	0.47	3.63	**2.87**
137	Aug-18	1.92	-0.23	4.07	1.53	-0.05	3.11	**1.35**
138	Sep-18	2.81	0.66	4.96	1.87	0.29	3.45	**3.01**
139	Oct-18	2.64	0.49	4.79	2.03	0.45	3.61	**2.25**
140	Nov-18	2.25	0.10	4.40	1.74	0.16	3.32	**2.33**
141	Dec-18	2.90	0.75	5.05	2.61	1.03	4.19	**2.35**
142	Jan-19	2.58	0.16	5.00	2.16	0.52	3.80	**1.56**
143	Feb-19	3.13	0.71	5.55	2.33	0.69	3.97	**1.53**
144	Mar-19	2.92	0.50	5.34	2.28	0.64	3.92	**3.30**
145	Apr-19	2.59	0.17	5.01	2.43	0.80	4.07	**3.68**
146	May-19	2.67	0.25	5.09	2.35	0.71	3.99	**2.50**
147	Jun-19	2.40	-0.02	4.82	1.83	0.19	3.47	**3.79**
148	Jul-19	2.29	-0.13	4.71	1.65	0.01	3.29	**2.15**
149	Aug-19	1.73	-0.69	4.15	1.41	-0.23	3.05	**3.37**
150	Sep-19	2.50	0.08	4.92	1.91	0.27	3.55	**2.76**
151	Oct-19	2.37	-0.05	4.79	2.00	0.36	3.64	**3.18**
152	Nov-19	2.12	-0.30	4.54	1.77	0.13	3.41	**3.75**
153	Dec-19	2.74	0.32	5.16	2.41	0.77	4.05	**2.21**
	Average	**2.62**			**2.12**			**2.66**
	Std. Dev.	**0.39**			**0.36**			**0.71**
	Minimum	**1.73**			**1.41**			**1.35**
	Maximum	**3.32**			**2.62**			**3.79**

In comparing the performance of the forecasts made for the monthly percentage of hospital discharges due to death using an ARIMA(0,0,01)(1,1,2)_12_ with drift model that did not consider a structural change and an ARIMA(0,0,0)(1,1,2)_12_ with drift model that did consider the structural change, the metrics listed in **Table 3** were obtained, and all the calculated error indices favored the ARIMA model that did not consider the structural change, namely, MAE = 0.63, RMSE = 0.81, MAPE = 25.89%, and MASE = 0.65.

**Table 3:** Forecast-accuracy metrics for the monthly percentage of hospital discharges due to death at the HRAEB.

Type of index	Evaluated error rate	Monthly percentage of discharges due to death	
		ARIMA(0,0,0)(1,1,2)_12_ not considering structural change	ARIMA(0,0,0)(1,1,2)_12_ considering structural change (t = 24)
Scale-dependent errors	MAE	0.63	0.74
	RMSE	0.81	0.95
Percentage error	MAPE	25.89%	25.75%
Scale-free error	MASE	0.65	0.76

MAE: Mean absolute error, RMSE: Root mean square error, MAPE: Mean absolute percentage error, MASE: Mean absolute scaled error.

## Discussion

In the study period (April 2007–December 2019), 3.41% (2,767/81,083) of hospital discharges from the HRAEB were due to death, which annually ranged from 2.50% to 5.77%, similar to the 4.10% identified by García. *et al*. [7] in a Spanish specialty hospital based on 24,194 episodes of hospitalization. These annual percentages are greater than the estimated 2% of hospital discharges per year due to death considering all Mexican SSA hospitals [3] and are also higher than the Mexican rate published in the journal *Salud Pública de México* in 1999 [2], which reported that 2.62% (35,891/1,469,161) of hospital discharges were due to death each year at the national level and 1.93% at the state level (Guanajuato, where the HRAEB is located) for 1999. There is little variability in the annual percentage of hospital discharges due to death at either the national or state level. The percentage of hospital discharges due to death at the HRAEB was higher than the national level and the state level (Guanajuato). This can be attributed to the fact that hospital discharges from the HRAEB correspond to patients with complex pathologies that require tertiary care, as suggested by Jiménez [8] in their study on the quantification of the quality of hospital services.

Regarding the monthly time series of the percentage of hospital discharges due to death in the HRAEB, a sudden change (structural change) in the trend of the time series was identified; this statistically significant change (p < 0.001) was detected at t = 24 (March 2009), which suggests that the trend of the series can be modeled by two linear segments: one increasing from April 2007 to March 2009 and another decreasing from April 2009 to December 2017. The factors that could influence the increasing trend and subsequent decreasing trend in the percentage of HRAEB hospital discharges due to death are as follows: First, the gradual hiring of specialized nursing staff in the HRAEB. Each year, the number of nurses (certified and specialized) has increased, since, as described by Aiken *et al*. [22], the number of nursing personnel as well as their level of professionalization affect hospital mortality. Leibson *et al*. [25] reaffirm the above facts and describe that a higher ratio of nurses per census bed decreases the mortality rate. Second, the constant increase in the volume of hospitalized patients in the first two years of HRAEB operation was associated with the habilitation and gradual certification of HRAEB services, consistent with the study by Taylor *et al*. [26], which described the association between the increase in the volume of patients and the increase in hospital mortality. Third, with the increase in the volume of surgical patients, approximately 80% of patients discharged from the HRAEB had undergone surgical procedures, which increases the likelihood of death from complex procedures, as detailed by Mcphee *et al*. [28], who identified the increase in the volume of surgeries as the main factor affecting mortality in one hospital.

The monthly predictions of the percentage of hospital discharges due to death from January 2018 to December 2019 were estimated by fitting seasonal ARIMA models, similar to the method of Fang *et al*. [12], but with and without consideration of the structural change identified by Chow’s test [31]. In comparing the performance of the predictions of both models with the actual percentages of hospital discharges due to death in that period (test dataset), the four calculated error metrics (MAE, RMSE, MAPE, MASE) favored the predictions made with the model that did not consider structural change. In our models, the estimated MAPE errors of approximately 25–30% may seem high but are because the values of the percentage of discharges for death were small (0–10%), which indicates that MAPE is not a good criterion for choosing the best model in this case, as described by Hyndman and Koehler [40]. The MAE and RMSE error indices are frequently used to determine which model generates the best predictions, perhaps due to their ease of calculation and interpretation, as observed in the study by Liu *et al*. [43]. However, a study by Hyndman [39] suggests that MASE is the best error metric to evaluate predictions in time series, such as ours, detailing that MASE values less than 1 indicate more accurate predictions. In our study, the two models constructed to predict the percentage of discharges due to death generated MASE values less than 1, but the model that did not consider structural change had a lower MASE value, suggesting that it is a more accurate model.

In future work, our data will allow us to evaluate various types of models (cubic, ARIMA, joint point), as Fang *et al*. did [12], and it will be necessary to identify the services that have the greatest influence on hospital mortality. It is necessary to study the percentage of hospital discharges due to death, conditioned on medical services in the HRAEB, and perform an analysis similar to that of Andrews *et al*. [9] that conditions mortality by specific diagnoses and procedures. In addition, the trends in mortality related to the specializations offered at the HRAEB could be specified, similar to what was described by Gonzaga *et al*. [44] in patients with breast cancer, Segura *et al*. [11] in patients with tuberculosis, and Fang *et al*. [12] in patients with liver cancer, to name a few.

Finally, the study has several limitations. First, since this was a retrospective study, causality could not be inferred. Second, as a secondary database was used, there could be minor capture errors inherent to the source itself. Third, since it was a single-center study in a tertiary care hospital, the results cannot be generalized to hospitals of other levels of care, although the analysis method described for analyzing the time series of mortality could be implemented to identify structural changes in hospitals of different levels of care.

## Conclusions

This study expands the knowledge on hospital mortality, showing that the overall percentage of hospital discharges due to death in a Mexican tertiary care hospital is 3.41%. Through the detection of a structural change (in March 2009) in the series of the monthly percentage of hospital discharges due to death, we identified that hospital mortality at the HRAEB can be described by a growing trend from its opening (April 2007) to March 2009 and by a decreasing trend from April 2009 to December 2019, in addition to seasonal behavior. Our study suggests that the seasonal ARIMA models constructed and evaluated with their respective prediction intervals are an adequate tool for future monitoring of the monthly percentage of hospital discharges due to death, allowing us to detect observations that depart from the described trend and identify future structural changes.

Despite this was a retrospective single-center study based on a secondary database, all this information generated and its methodology can be used to improve decision-making and resource management to reduce the rate of deaths in both HRAEBs and other health institutions. Further research efforts could be also devoted to identifying the medical services that have the greatest influence on hospital mortality via various types of mathematical models.
